# Skin-specific knockdown of hyaluronan in mice by an optimized topical 4-methylumbelliferone formulation

**DOI:** 10.1080/10717544.2021.1886376

**Published:** 2021-02-19

**Authors:** Emily H. Steen, Walker D. Short, Hui Li, Umang M. Parikh, Alexander Blum, Natalie Templeman, Nadine Nagy, Paul L. Bollyky, Sundeep G. Keswani, Swathi Balaji

**Affiliations:** a Department of Surgery, Division of Pediatric Surgery, Laboratory for Regenerative Tissue Repair, Texas Children’s Hospital and Baylor College of Medicine, Houston, TX, USA; b Department of Medicine, Division of Infectious Diseases, Stanford University School of Medicine, Stanford, CA, USA

**Keywords:** Hyaluronic acid, hyaluronan, inhibition, response surface methodology, genetic algorithm, topical, 4-methylumbelliferone (4-MU)

## Abstract

Hyaluronan (HA) is abundant in the skin; while HA can be synthesized by the synthases (HAS1-3), HAS2 is the leading contributor. Dysregulation and accumulation of HA is implicated in the pathogenesis of diseases such as keloid scarring, lymphedema and metastatic melanoma. To understand how HA synthesis contributes to skin physiology, and pathologic and fibrotic disorders, we propose the development of skin-specific HA inhibition model, which tests an optimal delivery system of topical 4-methylumbelliferone (4-MU).

A design-of-experiments (DOE) approach was employed to develop an optimal 4-MU skin-delivery formulation comprising propylene glycol, ethanol, and water, topically applied to dorsal skin in male and female C57BL/6J wildtype mice to determine the effect on HAS gene expression and HA inhibition. Serum and skin samples were analyzed for HA content along with analysis of expression of HAS1-3, hyaluronidases (HYAL 1-2), and KIAA1199.

Using results from DOE and response surface methodology with genetic algorithm optimization, we developed an optimal topical 4-MU formulation to result in ∼70% reduction of HA in dorsal skin, with validation demonstrating ∼50% reduction in HA in dorsal skin. 4-MU topical application resulted in significant decrease in skin HAS2 expression in female mice only. Histology showed thicker dermis in male mice, whereas female mice had thinner dermal layer with more adiposity; and staining for HA-binding protein showed that topical 4-MU resulted in breakdown in HA.

Our data suggest a topical 4-MU formulation-based dermal HA inhibition model that would enable elucidating the skin-specific effects of HA in normal and pathologic states.

## Introduction

Hyaluronan (HA), a large extracellular glycosaminoglycan comprised of repetitive glucuronic acid and N-Acetylglucoasamine (GlcUA-GlcNAc) disaccharides, is ubiquitous in vertebrate tissues. Approximately half of the total body HA content is in the skin under physiologic conditions, both in the dermis, where it is primarily produced by dermal fibroblasts, and in the epidermis, with the proliferating basal regions containing the majority (Tammi et al. 1988; Fraser et al. 1997; Papakonstantinou et al. 2012). In unwounded skin and in both normal and keloid scars, the dermis, particularly the papillary dermis, has repeatedly been shown to contain much higher levels of HA than the epidermis, where HA appears to be produced micro-locally by synthases specific to each layer (Meyer et al. 2000). Congruent with that, and despite the physical proximity of dermis and epidermis, HA appears to have a distinct function in each location. Epidermal HA is generally considered the source of skin hydration, while dermal HA appears to provide structural integrity in conjunction with collagen and elastic fibers. In addition to these location-specific roles, the HA-rich extracellular matrix plays a myriad of homeostatic roles in the skin as a whole, including barrier function and the regulation of growth factors, cytokines, and signaling essential for cellular proliferation, immunity, and wound healing (Day and de la Motte 2005). However, many chronic and inflammatory dermatitides are associated with pathologic tissue remodeling accompanied by elevated levels of HA, with perhaps the most notable being keloid scarring (Meyer et al. 2000). Additionally, HA has become increasingly recognized as an active contributor to cancer-promoting processes, like metastasis in melanoma (Toole et al. 2002). As HA is synthesized locally, extruding out of its parent cell directly into the extracellular space, a modality of skin-specific inhibition of HA synthesis would be a novel and advantageous tool for research of its role in physiologic wound healing and homeostasis, as well as its role in pathologic disease progression and treatment of these and other diseases such as lymphedema (Meyer et al. 2000).

The functional and homeostatic characteristics of HA are not only determined by quantity or organization into an extracellular matrix, but also by its molecular weight. High molecular weight HA (HMW-HA) predominates in the steady-state condition in skin, and is considered anti-inflammatory and anti-fibrotic (Bollyky et al. 2007). In contrast, the size profile skews toward low molecular weight HA (LMW-HA) in inflammatory conditions, acting as a damage-associated molecular pattern (DAMP). HA is produced by three hyaluronan synthases HAS1,2, and 3. HAS1 and HAS2 produce very high molecular weight HA (900 KDa and above), while HAS3 produces lower molecular weight HA (500KDa) (Bollyky et al. 2012; Balaji et al. 2017). Though encoded on different chromosomes and with tightly-regulated spatiotemporal expression patterns, HAS1-3 are well-conserved enzymes with a high degree of functional redundancy due to their crucial role during embryonic development. The degradation of HA is regulated by hyaluronidases (HYAL), with HYAL1 and HYAL2 considered key enzymes for HA degradation. KIAA1199 has been shown to have a role in binding and depolymerization of HA in skin fibroblasts and its expression by dermal fibroblasts has a key role in HA degradation in normal skin (Yoshida and Okada 2019). In the skin, HAS1 predominates in epidermal keratinocytes, while HAS2 is the primary isoform in dermal fibroblasts, and the presence and relative quantity of all three of these enzymes appear to play critical roles in keloid formation and skin cancer (Edward et al. 2010). Notably, knockout of HAS2 is embryonic lethal, underscoring the importance of this family of enzymes. In addition, the pathologic hypermobility of epidermal keratinocytes that contributes to the “over-healing” of wounds in keloid formation has been attributed to HAS2 overexpression, and similarly, the role of HAS2 in promoting keratinocyte migration appears to be involved in melanoma metastasis (Edward et al. 2010; Sidgwick et al. 2013; Supp et al. 2014). Unfortunately, HAS deletion has not been effectively modeled to this point: shRNA knockdown of the HAS family of enzymes has been poorly reproducible, and pure HAS knockout murine models have not been successful or standardized. A dual HAS1/HAS3 knockout (Kessler et al. 2015) combined with an inducible HAS2 knockout has previously been developed but has not been efficiently repeated. Given this lack of an animal model, other non-genetic means of HAS knockdown and HA inhibition must be sought.

One of the most well-known inhibitors of HA synthesis is 4-methylumbelliferone (4-MU). A coumarin derivative, 4-MU has been shown to experimentally reduce HA levels both *in vivo* and *in vitro* (Kakizaki et al. 2004; Kultti et al. 2012; Nagy et al. 2015). Though many of its functions are still yet unidentified, three primary mechanisms of action are well described: first, it acts as a competitive substrate of UDP glucuronosyltransferase (UGT), a necessary enzyme in the creation of the dimer unit (GlcUA-GlcNAc) that comprises the HA molecule. Second, 4-MU has been reported to reduce concentrations of UDP-GlcUA *in vitro* via binding directly to glucuronic acid (Kultti et al. 2012). Finally, 4-MU is also capable of reducing mRNA expression levels of HAS2 (Kultti et al. 2012). Many groups have developed strategies for intravenous administration of 4-MU or oral administration with 4-MU added into the mouse chow (Kuipers et al. 2016), which have proven very useful for understanding the role of HA in several disease processes, but the systemic side effects of such treatments are either unknown, strongly confounding, or undesired.

In this study, we demonstrate the development of a skin-specific means of HA inhibition using an optimal topical delivery system of 4-MU. This transdermal formulation uses a combination of propylene glycol, ethanol, and water which are routinely used components in topical drug delivery. To optimize 4-MU delivery and efficacy as demonstrated by HA reduction, we employed a design of experiments (DOE) with response surface methodology (RSM) for modeling of the process parameters coupled with genetic algorithm (GA), a powerful computational tool that allows for the optimization of dose of components. The optimized formulation was then successfully validated on adult mice, along with the analysis of its effect on skin histology and HA levels, HAS1-3, and HYAL1-2 and KIAA1199 expression in dorsal murine skin.

## Materials and methods

### Animal studies

Male and female C57BL/6J mice were used in these studies. All animals were bred and maintained under specific pathogen-free conditions with free access to food and water in the animal facilities at Texas Children’s Hospital Feigin Center (Houston, TX, USA). Animal protocols were approved by the Institutional Animal Care and Use Committee (IACUC) at Baylor College of Medicine.

### Development of topical formulation

#### Design of experiments/central composite design

**Figure 1.**
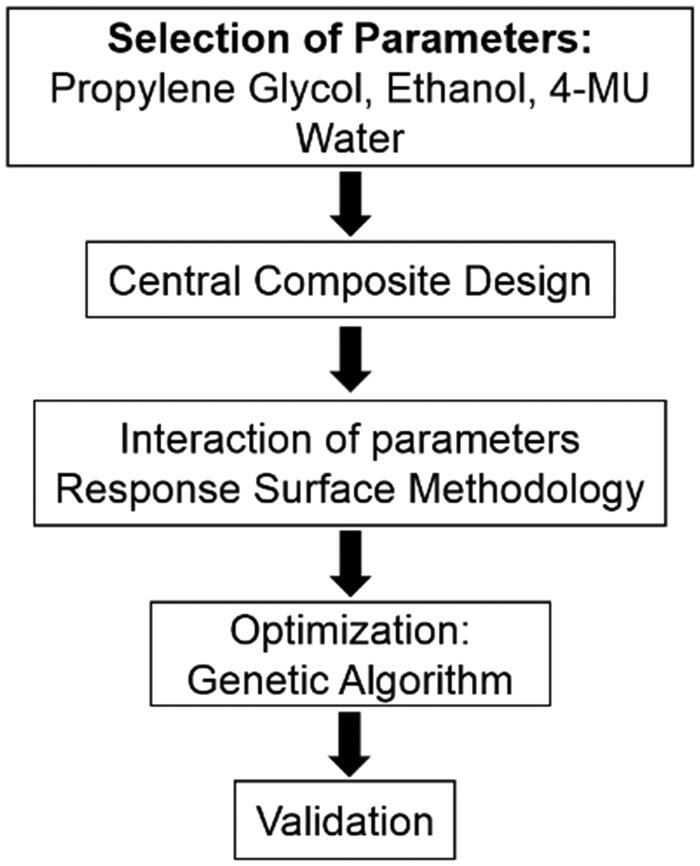
Flow chart of experimental design. Parameters for the development of topical formulation for 4-MU delivery were selected based on standard pharmacology of topical applications. Central composite design was used to define formulation parameters and local optima. Response surface methodology was used to generate a regression equation that predicts HA decrease, which was optimized using genetic algorithm. The optimal formulation was then validated *in vivo* for its effect on HA levels.

A topical 4-MU formulation, based on standard pharmacology of topical applications was developed using propylene glycol (PG), ethanol (EtOH) and water with 4-MU as our active element. Based on the literature review, we first defined the minimal and maximal concentrations of each of the components in the topical formulation. In order to optimize the percentage of each of these components in the formulation to obtain maximal HA reduction in skin, an initial design of experiments (DOE) was performed formulated with the above 3 parameters with minimum and maximum concentrations (boundary conditions) for each parameter as follows: PG (0 and 60%), EtOH (0 and 60%), and 4-MU (0 mM and 2 mM) (Table 1), and remaining volume made up with water. Central Composite Design predicted 15 different formulations with varying does of each of the individual components (Table 2), each of which were applied to the backs of the initial test cohort of mice to study the effect of each combination on skin HA levels at the completion of the time course of topical application. RSM was used for modeling the process and studying the interaction between parameters for the reduction of HA. The regression equation was then used as objective function for process optimization in GA (Figure 1). The predicted optimal formulation was then validated in a second cohort of mice. DOE and RSM are commonly used to develop optimal concentrations of products used in the biotechnology field, particularly with agricultural and food engineering. The utility of these methods lies in the ability of these powerful computational tools to extrapolate a wide range of responses from a limited number of biologic experiments, once maxima and minima concentrations of each of the components that go into the formulation are defined based upon industry standards or literature.

**Table 1.** Boundary conditions of selected parameters for the design of experiments.

PG (0-60%*v/v*)	0%	15%	30%	45%	60%
EtOH (0-60%*v/v*)	0%	15%	30%	45%	60%
4-MU (0–2 µM)	0 µM	0.5 µM	1 µM	1.5 µM	2 µM

**Table 2.** Central composite design for the initial development of topical 4-MU formulation.

Mouse No.	PG(%*v/v*)	EtOH(%*v/v*)	4-MU(µM)
1	15	15	0.5
2	45	15	0.5
3	15	45	0.5
4	45	45	0.5
5	15	15	1.5
6	45	15	1.5
7	15	45	1.5
8	45	45	1.5
9	0	30	1
10	60	30	1
11	30	0	1
12	30	60	1
13	30	30	0
14	30	30	2
15	30	30	1
16	30	30	1
17	30	30	1
18	30	30	1
19	30	30	1
20	30	30	1

#### Response surface methodology (RSM)

RSM is an empirical modeling technique used to evaluate the relationship between a set of controllable experimental factors and observed results (Bhattacharya et al. 2011). This optimization process involves three major steps: (i) performing statistically designed experiments, (ii) estimating the coefficients in a mathematical model and (iii) predicting the response and checking the adequacy of the model (Box and Behnken 1960). RSM allows us to use DOE and central composite design (CCD) to approximate the values of our explanatory variables (PG, EtOH, and 4-MU) which will give the greatest reduction in dermal HA. This design was applied to our study using Minitab^®^, with three variables at four levels. The three significant variables can be approximated by the quadratic model equation as follows:

(1) $$Y\;={\;k}_{0}+{\text{ k}}_{a}A\;+{\text{ k}}_{b}B\;+{\text{ k}}_{c}C\;+{\text{ k}}_{\text{aa}}A^{2}+{\text{ k}}_{\text{bb}}B^{2}+{\text{ k}}_{\text{cc}}C^{2}+{\text{ k}}_{\text{ab}}{\text{AB}+k}_{\text{ac}}{\text{AC}+k}_{\text{bc}}\text{BC}$$



where Y is the predicted response; k_0_ is a constant; k_a_, k_b_, k_c_ are the linear coefficients; k_ab_, k_ac_, k_bc_ are the cross-coefficients; k_aa_, k_bb_, and k_cc_ are the quadratic coefficients. A total number of 15 experimental formulations were necessarily carried out to estimate the optimal reduction in HA (Table 2). 150 µl of topical drug formulation was massaged into the dorsum of experimental mice (both male and female at 6–10 weeks of age) for 7 days, after which animals were euthanized and dorsal skin was excised. Skin tissue was homogenized and the content of HA was evaluated using HA ELISA as described below. For all the experimental formulations, at least 2 mice were tested and the average value of HA level in the skin after application of the formulation was obtained, except at the midpoint composition where the DOE included several mice being tested already. Data were analyzed using Minitab^®^ and Design Expert 7^®^ programs including analysis of variance (ANOVA) to determine the interaction between the variables and the response. The quality of the fit of this model was expressed by the coefficient of determination (*R*^2^) and studentized plot in the same program.

#### Optimization using genetic algorithm (GA)

The genetic algorithm is a stochastic optimization tool that belongs to the class of evolutionary algorithms that use techniques inspired by the Evolutionary theory of Darwin such as inheritance, crossover, mutation, and natural selection. The genetic algorithm is typically implemented in the form of computer simulations where a population of abstract representations (called chromosomes) of candidate solutions (called individuals) to an optimization problem evolves gradually toward better solutions. Our input variables (4-MU, PG, and EtOH) were treated like genes “embedded” in a chromosome. The fitness function (Equation (1)) obtained from the RSM was used as an input in GA, as previously described (Bhattacharya et al. 2013), to create the initial population of the solutions (HA reduction) and genetic variants (of the parameters) in the offspring using genetic operators (crossover and mutation), evaluation of these candidate solutions in light of feasibility, and finally evaluating the altered offspring (parameter combination variants). These randomizations in the GA were more likely to overcome the constraints of local optima, thereby attaining the global optima for the reduction of HA. Minitab 13^®^ and Matlab^®^ (version 9) softwares were used in the present investigation. Optimtool^®^ add-on program was used for Matlab^®^ for the optimization using Genetic Algorithm.

### Research design

After the optimized concentrations of the different parameters were determined as above, a topical 4-MU treatment application was formulated using the optimal concentrations of 4-MU, propylene glycol (PG), ethanol (EtOH), and deionized water as the vehicle. Validation of this formulation on skin HAS1-3 and HYAL1-2 and KIAA1199 expression and HA inhibition was performed, with experimental groups consisting of topical 4-MU treatment application, topical sham treatment application, and control/untreated animals. One day prior to treatment, the skin of the mice was shaved and Nair™ (Church & Dwight, Ewing, NJ, USA) was used to defoliate the dorsum of mice. On the day of treatment, skin was shaved and scrubbed with alternating betadine and 70% isopropyl alcohol three times. 150 µl of topical drug formulation was massaged into both flanks of experimental mice twice daily for 7 days and the area of application was covered with non-adhesive OpSite^®^ film dressing. These mice received normal chow *ad libitum* (*n* = 6 mice, 3 females and 3 males at 7–8 weeks were included). The sham control set of mice underwent the same treatment using a mixture of only PG, EtOH, and water (without 4-MU) (*n* = 6 mice, 3 females and 3 males at 7–8 weeks were included). Following topical applications, animals were euthanized, and skin and serum were collected, and HAS1-3, HYAL1,2 and KIAA1199 and HA levels were determined.

To establish baseline HA content in murine skin, we harvested dorsal and ventral skin from untreated male and female C57BL/6J at 1, 4, 6, 8, 15, and 24 weeks (*n* = 30 total, 4–6 mice, with equal number of male and female mice included in the analysis at each age). These mice were maintained in standard lab conditions and received standard chow ad libitum at all time points.

#### HA extraction and quantification

Mouse skin samples were weighed and homogenized in freshly made, pH optimized lysis buffer comprised of 0.15 M Tris base, 0.15 M NaCl, 0.01 M CaCl_2_, 5 mM deferoxamine, and 20 U/ml *Streptomyces griseus* protease (Sigma) in a ratio of 15 µl buffer per 1 mg of tissue. Each sample was digested at 55 °C for 16 hours on a shaker at 1400 rpm. To stop digestion, the protease was denatured on a hot plate at 95 °C for 10–15 minutes. The supernatant was collected from each tissue sample. HA quantification in skin and serum samples was then performed using an ELISA-like sandwich protein binding assay as per the commercial manufacturer's instructions (Corgenix). Briefly, the assay uses microwells coated with highly specific HA binding proetin (HABP) to capture HA from the test samples. 50 μl of the samples or standards were pipetted into the HABP coated microwells and incubated for 1 hr, and an enzyme conjugated version of HABP was used to detect HA. The absorbance of each sample was measured at 450 nm using a plate reader and optical density was blanked against reagent reference. HA concentration in the samples was measured by comparing absorbance againt standard curve. The HA values were normalized to total protein determined from the undigested homogenate using Coomassie plus assay.

#### Coomassie plus assay

Total protein quantification was performed using Coomassie Plus assay as per the manufacturer's instructions (Thermo Scientific). Briefly, 10 µl of the samples or albumin (BSA) standards were pipetted into a 96 well plate in duplicate. 300 µl of Coomassie Plus reagent was added to each well and placed on a plate shaker protected from sunlight for 5–10 minutes. The absorbance of each sample was read at 595 nm on a spectrophotometric plate reader and blanked against water. Standard dilutions were used to derive a four-parameter best-fit standard curve to calculate sample protein concentration (µg/ml). HA-ELISA values were then normalized against total protein content of the sample for the purposes of this study. No Coomassie assay was performed for serum samples.

#### Histology

Skin tissue after different treatments were harvested, fixed in 10% neutral buffered formalin and paraffin embedded. 5 µm thick sections were cut and mounted onto slides. Slides were deparaffinized and rehydrated to TBS following standard protocol. Sections were blocked in TBS containing 10% fetal bovine serum (FBS) + 1% BSA, followed by staining with biotinylated HABP with 0.4 μl of (0.5 μg/μl stock) (EMD Millipore, 385911) HABP/100 μl + 1% BSA in PBS applied overnight. Slides were then developed with Streptavidin and DAB with Hematoxylin counterstaining and covered using Aquatex. Parallel sections that went through the whole procedure, with the only exception of HABP application, served as no staining controls, while sections that were pretreated for an hour with 1 mg/ml hyaluronidase (Worthington Biochem, LS002594) at 37 °C served as negative controls to demonstrate the specificity of the staining.

Histology slides were imaged with Leica DM 2000^®^ with Leica application Suite X^®^ version 3.0.4.16529. 40× magnification images were taken and the skin tissue was qualitatively analyzed for changes in HA staining patterns in the epidermis, dermis, and fat tissue.

#### Real-time quantitative PCR

Skin tissue were lysed in 350 ml Buffer RLT (Qiagen, Valencia, CA, USA). Total RNA was isolated using the RNeasy Micro Kit (Qiagen). cDNA was synthesized from 5 ng of RNA by using the High-Capacity cDNA Reverse Transcription Kit (Applied Bio- systems, Foster City, CA, USA) following manufacturer protocols. SYBR green assays were designed to span intron/exon boundaries. Oligonucleotides were aligned against the mouse genome by Primer-BLAST (http://www.NCBI.org) to ensure specificity. Gene expression was assayed in triplicate using 1/40 of the cDNA template and 300 nM of forward and reverse primers in a 20 l Power SYBR Green PCR Master Mix reaction in a StepOne-Plus Real-Time PCR System (Applied Biosystems). Gene expression was normalized to mouse GAPDH gene expression. Relative expression values were calculated using the DDC_t_ method (Pfaffl 2001). Oligonucleotide primer sequences for HAS1, HAS3, and HYAL1,2 and KIAA1199 are listed in Table 3. Mouse HAS2 (Bio-Rad, qMmuCID0024398), and HYAL1 (Bio-Rad, qMmuCED0040215) primers were purchased from Bio-Rad Laboratory.

**Table 3.** Primer sequences for quantitative PCR validation.

Oligonucleotide	Sequence, 5′–3′	Amplicon size (bp)	Intron spanning
Mo HAS1 2 F	CATGGGCTATGCTACCAAGT	77	3251
Mo HAS1 2 R	TCAACCAACGAAGGAAGGA		
Mo HAS2	[Bio Rad Primer]		
Mo HAS3 1 F	CAGTGGACTACATCCAGAGGTG	73	2345
Mo HAS3 1 R	ACTCGAAGCATCTCAATGGT		
Mo HYAL1	[Bio Rad Primer]		
Mo HYAL2 1 F	TCTTCACGCGTCCCACATA	79	421
Mo HYAL2 1 R	GCACTCTCACCGATGGTAGA		
Mo KIAA1199 1 F	CTCAGCTGAAGACAAAAGAC	77	1F split by 187-bp intron
Mo KIAA1199 1 R	ATTCCGAAGGTGGAAGAAG		
Mo GAPDH F	GCTGGAGAAGGTTTGTGCG	100	182 and 252
Mo GAPDH R	AGTGATTCTCAAAGTCTTGGTAGGC		

F: forward; R: reverse.

#### Statistical analysis

Statistical analysis of data was performed using ANOVA, followed by Bonferroni post-hoc tests or student’s *t*-test when appropriate. Data are expressed as means ± SD. A value of *p*<.05 was considered significant.

## Results

### Initial development of topical 4-MU formulation

The 15 experimental combinations (Table 2) established in the DOE for the development of topical 4-MU formulation were applied to the dorsal skin of male and female 6- to 10-week-old WT C57BL/6J mice. HA ELISA-like assay was performed on harvested skin samples from those mice (normalized to total protein in the skin), and results for each condition are shown in Table 4.

**Table 4.** Experimental vs. predicted values from model.

Mouse No.	PG (%*v/v*)	EtOH (%*v/v*)	4-MU (µM)	Dorsal HA (ng)	Protein (µg)		Normalized value (ng/µg)	Predicted value (ng/µg)	Residual (ng/µg)
1	15	15	0.5	37674.1		115.342	326.629	273.652	5.2978
2	45	15	0.5	56566.0		223.722	382.84	453.737	−7.0897
3	15	45	0.5	41951.1		130.050	322.576	347.292	−2.4715
4	45	45	0.5	46465.7		130.962	354.802	425.022	−7.022
5	15	15	1.5	52051.7		150.900	344.94	334.079	1.0861
6	45	15	1.5	66659.6		238.738	329.216	363.859	−3.4643
7	15	45	1.5	78266.9		223.722	349.839	338.3	1.1539
8	45	45	1.5	81607.7		532.033	153.388	265.724	−11.2336
9	0	30	1	22041.9		110.689	199.132	254.143	−5.501
10	60	30	1	50907.4		106.943	476.021	361.652	11.4368
11	30	0	1	59071.6		168.031	351.551	360.38	−0.8829
12	30	60	1	65346.9		161.721	404.072	335.885	6.8187
13	30	30	0	50718.0		142.767	535.248	508.5	2.6748
14	30	30	2	45626.9		103.172	442.239	409.629	3.261
15	30	30	1	77065.2		262.719	293.336	285.737	0.7599
16	30	30	1	70611.3		217.222	325.065	285.737	3.9328
17	30	30	1	51033.8		171.691	297.242	285.737	1.1505
18	30	30	1	17444.0		65.3399	266.973	285.737	−1.8764
19	30	30	1	18278.7		59.8604	305.355	285.737	1.9618
20	30	30	1	51160.5		179.002	285.809	285.737	0.0072

The topical combination without 4-MU (formulation no.13) had the highest skin HA levels (535.248 ng/µg protein) in these cohort of animals. The last 6 animals (animals 15–20) were experimental replicates, administered with a topical formulation at the midpoint of the CCD; all 6 resulted in a consistent degree of HA reduction in the skin.

From these experimental values, a regression equation (Equation (2)) was created that included all 3 parameters as variables.

(2) $$\text{HA }\left(\text{ng/µg}\right)=\;22.95\;+\;0.87\left(\text{PG}\right)\;+\;0.12\left(\text{EtOH}\right)\text{ + 17}\text{.64}\left(\text{4–MU}\right)\text{ + 0}{\text{.01(EtOH}}^{\text{2}}\text{)}\text{+}\text{ 17}{\text{.33(4–MU}}^{\text{2}}\text{) – 0}\text{.01}\left(\text{PG}\right)\left(\text{4–MU}\right)\text{ – 0}\text{.23(EtOH}\left(\text{4}-\text{MU}\right)$$

when these parameters were varied to match the 15 experimental conditions, the predicted HA values strongly resembled those of the actual HA values, with the greatest residual being 11 (ng/µg protein) and an *R*^2^ coefficient of .832 (83.2%), demonstrating considerable goodness of fit. Taken together, these observations validate both the regression equation in how well it approximates the real, as well as the prediction of the most effective 4-MU formulation.

**Figure 2.**
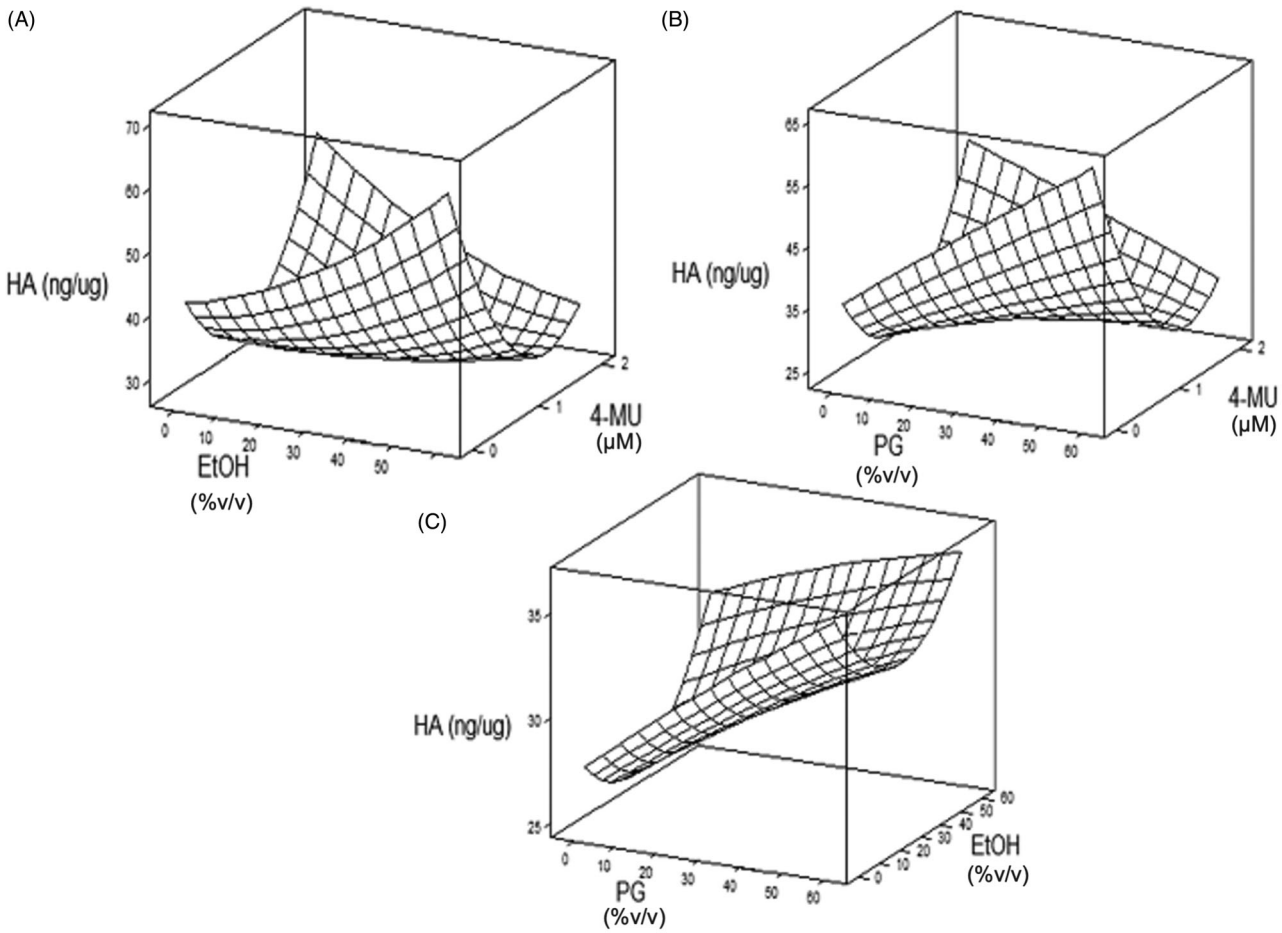
Response surface methodology plots. (A) HA response to varied levels of EtOH and 4-MU. Decreased HA noted with low EtOH and high 4-MU, as well as high EtOH and low 4-MU. (B) HA response to varied levels of PG and 4-MU. Decreased HA noted with low PG and low-moderate 4-MU. (C) HA response to varied levels of PG and EtOH. Decreased HA noted with low-moderate EtOH and low PG.

The RSM plots (Figure 2) were critically analyzed to understand the interaction of the manipulated parameters on HA reduction. From the EtOH and 4-MU response surface plot (Figure 2(A)), decreased HA was observed with low levels of EtOH and moderate levels of 4-MU. Elevated HA was noted when high 4-MU was paired with low EtOH and low 4-MU paired with high EtOH. In the PG and 4-MU plot (Figure 2(B)), decreased HA was noted with low proportion of PG and moderate levels of 4-MU, with HA level responses seen in a similar pattern as EtOH and 4-MU with elevated HA noted when high 4-MU was paired with low EtOH and low 4-MU paired with high EtOH. Higher proportion of 4-MU did not necessarily result in greater decrease in HA, as we posit that the ethanol and PG also have a role in the penetration and availability of the active ingredient 4-MU in the dermis where HA is in abundance in the skin. The PG and EtOH plot (Figure 2(C)) demonstrate decreased HA with low-moderate EtOH and low PG, with elevated HA associated with high PG in both extremes of low and high EtOH.

Ultimately, these results concluded with the determination of an optimized formulation of 0.82 mM 4-MU in 16.32% volume/volume% (*v/v*) PG, 15.71% (*v/v*) EtOH, and remaining 67.97% (*v/v*) water predicted to effect ∼70% HA decrease in the skin.

### Validation of topical 4-MU treatment

**Figure 3.**
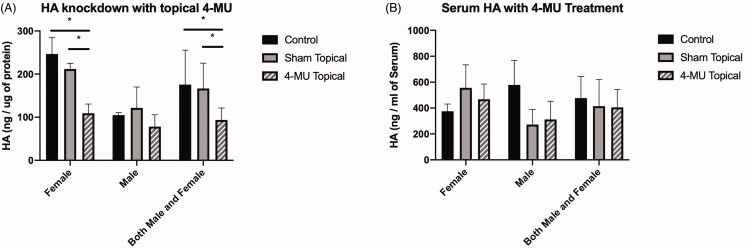
Validation of topical 4-MU formulation. (A) HA levels in dorsal skin significantly reduced between control and topical 4-MU application, as well as between sham and topical 4-MU treatment, as determined by ELISA-like assay. HA in females treated with 4-MU formulation was observed to be lower than females treated with sham or no treatment. No significant difference between treatments is seen in males. (B) Serum HA levels measured by ELISA-like assay were not significantly different between treatment groups in either sex. *n* = 6–8 (3–4 male and 3–4 female) mice per treatment group. Bar plots show average ± standard deviation. * p<.05

Analysis of HA levels in the skin of mice that were treated with the predicted optimal 4-MU topical formulation (*n* = 6 mice, 3 males and 3 females, 7–8 weeks of age) showed a significant reduction as compared to the untreated group (*n* = 8 mice, 4 males and 4 females, 7–8 weeks) (93.63 ± 27.85 ng/µg protein vs. 175.70 ± 79.98 ng/µg protein, *p*<.05). While the predicted reduction in HA was ∼70%, the data obtained from the mice with equal number of males and females grouped together in each treatment showed ∼50% reduction in HA expression in dorsal skin. However, we observed differences in the effect of 4-MU topical treatment among female and male mice, with female mice showing ∼60% reduction (*p*<.01), as compared to male mice which showed ∼26% reduction (*p*=.11) in HA levels as compared to their respective untreated controls. Sham treatment (*n* = 6 mice, 3 males and 3 females, 7–8 weeks) did not result in a significant decrease in skin HA levels compared to untreated (93.63 ± 27.85 ng/µg protein vs. 166.7 ± 58.68 ng/µg protein, *p*<.05). However, we observed a slight reduction in the HA levels in the skin of female mice with sham topical treatment as compared to their untreated controls. (Figure 3(A)). In spite of the differences in HA content seen at the level of the skin in the different treatment groups, serum HA levels were not significantly different in any group compared to untreated (Figure 3(B)).

### Changes in gene expression of hyaluronan synthases and hyaluronidases

**Figure 4.**
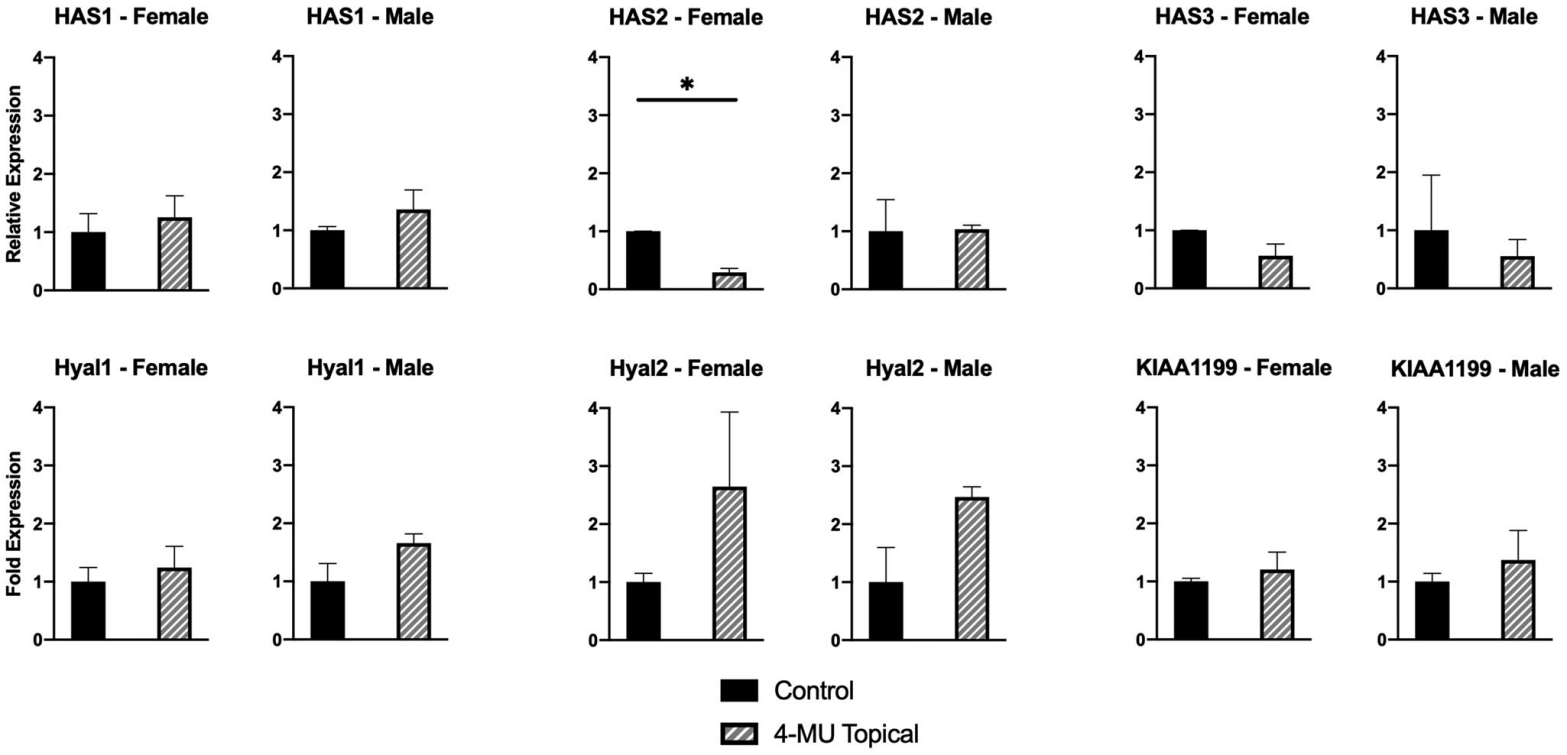
Hyaluronan synthase and hyaluronidase expression in murine dorsal skin. Topical 4-MU treatment only significantly decreased expression of HAS2 in female mice as shown by qRT-PCR analysis of dorsal skin. No significant difference in HAS2 expression was seen in male mice. *n* = 6-8 (3–4 male and 3-4 female) mice per treatment group. Bar plots show average ± standard deviation. * p<.05

Because HA content in any tissue is a product of the balance of synthesis and degradation, changes in gene expression of HA synthases and hyaluronidases with topical 4-MU treatment were assessed. These results are shown in Figure 4. Skin HAS2 expression was significantly downregulated in female mouse skin after topical 4-MU treatment, as compared to untreated female mice, while HAS1 and HAS3 expression was not significantly changed between treated and untreated animals. This was not seen in male mice: none of the three synthases were downregulated in response to topical 4-MU treatment in male mice. No difference was seen in HYAL1, HYAL2, or KIAA1199 expression between groups.

### HA staining pattern in the skin of topical 4-MU treated mice

**Figure 5.**
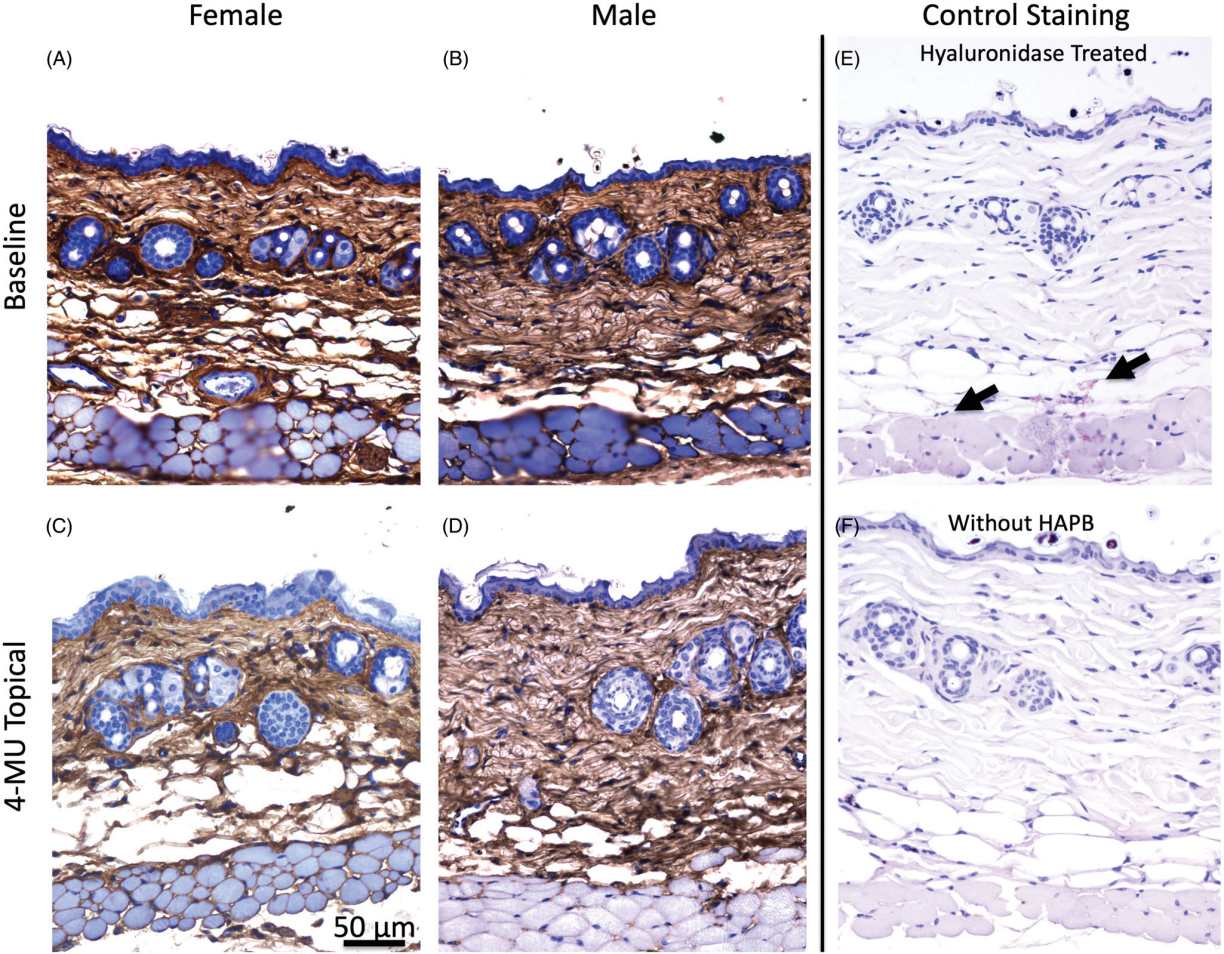
Qualitative analysis of histology in control and treated mice. (A–D) HABP stained representative 40× images of dorsal skin of baseline (top row) vs. topical 4-MU treated (bottom row) in female (left column) and male (right column) mice are shown. (A–B) Female mice have thicker epidermal layer and more adiposity at baseline, while male mice have a thicker dermal layer and less adiposity. (A–D) Treatment with topical 4-MU resulted in dermal matrix disorganization in both male and female mice compared to baseline. (E–F) Control staining of dorsal skin. (E) HABP staining following treatment with hyaluronidase. Arrows indicate areas of residual HA following hyaluronidase digestion. (F) Secondary antibody only staining without HABP.

We used HABP to probe for the effect of the topical 4-MU formulation on histologic staining pattern of HA in the skin. In the untreated mice, we found that HABP stained the dermis uniformly and with high intensity. Pretreatment of histologic sections with hyaluronidase prior to staining with HABP was done to ensure that we were accurately analyzing skin HA, and these sections were clear of any staining except in the residual connective tissues that were clearly cleaved with hyaluronidase, but not fully washed off from the histologic slides during the staining procedure. 7–8-week-old female mice were observed to have a thicker epidermal layer and more adiposity at baseline; male mice, on the other hand, had a thicker dermal layer and less adiposity. Representative images from our histological results are in Figure 5. There are multiple established differences in the architecture of normal male and female mouse skin; and our findings are in agreement with previous findings reported in the literature (Hanley et al. 1996; Azzi et al. 2005, 2006; Dao and Kazin 2007). We observed that treatment with topical 4-MU notably resulted in dermal matrix disorganization and a breakdown in the normal dermal HA pattern in both male and female mice relative to untreated controls, with no apparent exacerbation of this effect on the basis of sex.

### Skin HA content varies with age and sex

**Figure 6.**
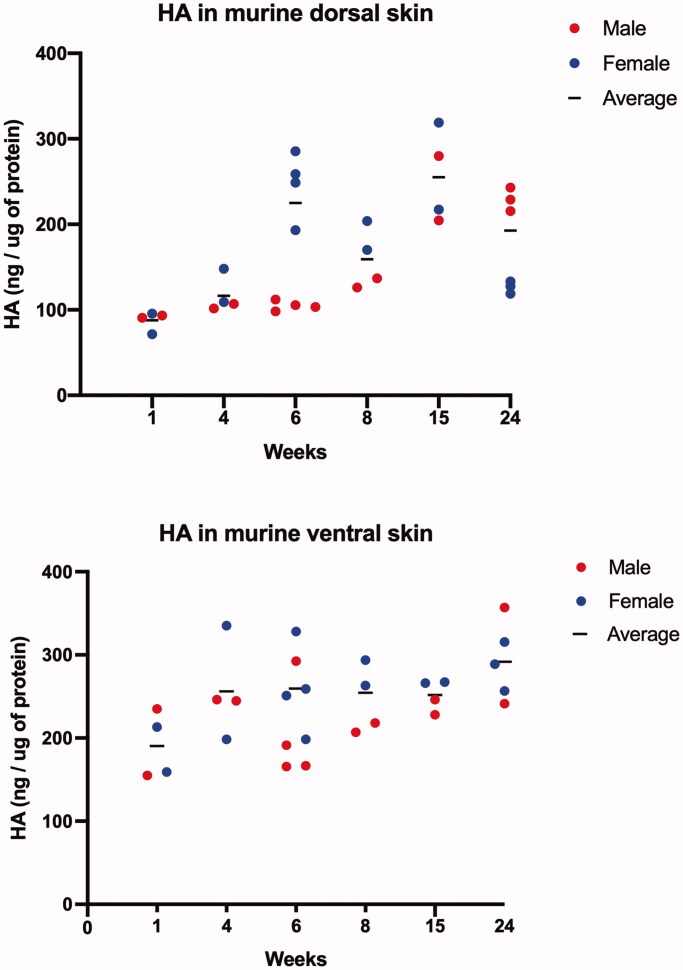
Analysis of baseline skin HA content at varying ages. (A) HA content in dorsal skin of mice, as measured with ELISA-like assay, is noted to progress with age, with dips in expression at 8 and 24 weeks of age, particularly in female mice. (B) Relatively constant level of HA is noted in murine ventral skin in both sexes. HA levels in each mouse skin are plotted, with red dots indicating male and blue dots indicating female mice. Black line represents average HA (ng/µg of protein) of both male and female mice. *n* = 30 mice (2–3 male and 2–3 female mice) per age group.

As validation experiments in male mice were not congruent with the predicted model between sexes, we sought to establish whether sex- and age-specific differences in baselines for HA levels in murine skin exist that would explain our observed differences in predicted vs. observed HA reduction. Dorsal and ventral skin from untreated mice were harvested. We chose six time points (1, 4, 6, 8, 15, and 24 weeks) for sample harvest, as ∼8 weeks is an established time point of adulthood at which they are predominantly used in the murine wound healing, fibrosis and other dermal diseases literature, while 6/15 weeks and 8/24 weeks are well-described in murine hair cycling (anogen and catogen phases, respectively) (Plikus and Chuong 2008). These results are shown in Figure 6.

In ventral skin, HA levels are largely consistent over time in both male and female mice. After weaning, however, HA levels in murine dorsal skin overall tends to increase in both male and female mice groups. In accordance with that overall finding, the dorsal skin of male mice showed a fairly consistent accumulation of HA over the time period followed, though this increase was not statistically significant from week to week. In contrast, levels of HA in the dorsal skin of female mice appeared closely temporally related to the known pattern of hair cycling in mice, with spikes in HA content at both 6 and 15 weeks (anogen phase) and apparent downregulation at 8 and 24 weeks (catogen phase). Estimation of correlation revealed low to moderate correlation between overall HA levels in both dorsal and ventral skin of combined sexes with age (Pearson Correlation Coefficient 0.364 and *p*=.0043). Correlation between HA in dorsal and age, and HA in ventral skin and age in combined sexes revealed similar low to moderate correlation (Dorsal skin: Pearson Correlation Coefficient 0.397 and *p*=.0296; Ventral skin: Pearson Correlation Coefficient 0.497 and *p*=.0051). When correlation between HA levels in skin and age were calculated separately for each sex, the analysis showed no significant positive correlation in female mice (Pearson Correlation Coefficient 0.131 and *p*=.488), but showed a moderate to medium correlation in male mice (Pearson Correlation Coefficient 0.618 and *p*=.0003).

## Discussion

In this study, we describe the development and testing of a novel topical 4-MU formulation that results in effective reduction of HA in the murine skin, while observing marked sex-based differences in the dermal response to topical 4-MU application. As we found HAS2 levels to be downregulated by topical 4-MU application; this synthase thus appears to be the most likely target in achieving these effects. With HAS1 as the main synthase involved in HA production by normal human epidermal keratinocytes and HAS2 as the primary isoform in dermal fibroblasts, it appears that these isoforms are differentially regulated by 4-MU topical application, a process which could have influenced our findings (Day and de la Motte 2005; Malaisse et al. 2014). Despite this, our work provides important insight into the mechanism of action and cellular players affected by 4-MU in the skin, as the current literature is disparate with mechanisms not completely elucidated (Malaisse et al. 2014). Some have shown that tissue injury stimulates an increase in HAS2 and −3 expression and thereby HA levels in the dermis: the physical action of application of a topical product or the product itself can initiate a confounding dermal reaction in mice, as in tape stripping experiments, or the apparently inactive constituents of the solution (PG, EtOH) may have functional side effects (Balaji et al. 2017; Garantziotis and Savani 2019). However, we found HAS2 expression to be downregulated, not upregulated, with no gross changes in mice skin by observation after topical 4-MU application in both male and female mice, making this possibility unlikely and not a major concern in our studies.

It is notable that our pilot studies of different topical 4-MU formulations for optimization were performed on mice at 6–10 weeks of age, a time frame in which the baseline HA content of murine dorsal skin is variable, particularly in female mice. Whereas the more definitive studies using the optimized topical 4-MU formula were performed at 7–8 weeks of age, where the HA levels are at a natural low at 8 weeks. This may explain why HA reduction was somewhat less effective in the validation experiments – as there was not as much baseline HA in the skin, we posit the natural nadir blunted the apparent knockdown efficacy. In addition, sex differences in mouse skin may have played a role in the apparent efficacy of the topical formulation. These differences have been well described by Azzi et al. using hormone therapy and gonadectomy and appear to be sex hormone receptor-dependent (Hanley et al. 1996; Azzi et al. 2005; 2006). This sexual dimorphism may play a role in the difference in overall efficacy of our topical drug in that female mice have higher adiposity and thinner dermal layer, to unknown pharmacokinetic effect. The differences in efficacy between female and male could also be attributable to the differences in baseline HA levels at the time point when topical 4-MU treatments were initiated, wherein untreated female mice had higher starting HA content in their dorsal skin compared to male skin at the same location and at the same age. Further, our design of experiments did not distinguish by sex of the mouse on which the formulation was tested. It is conceivable that the inherent differences in the makeup of skin between the sexes means that there is a differing optimal topical 4-MU formulation specific to male vs. female mice.

Currently, pressed chow is the gold standard for experimentally administering 4-MU to animals, but the need for a period of several weeks of “loading dose” to see an effect on HA levels in the serum—much less the skin—is a significant limiting factor (Kuipers et al. 2016). This long transition period needed before the appearance of its effects may be secondary to the fact that mice historically find 4-MU chow objectionable at first, even losing weight in the first days of their change in diet. Conversely, our optimized topical 4-MU formulation showed rapid and significant skin-specific effects after only one week of noninvasive application. In addition, the topical formulation delivers an almost 100-fold lower dose of 4-MU than the pressed chow (0.021 µg versus 250 mg daily, respectively). Since 4-MU’s mechanism of action is not yet fully elucidated, and accordingly its side effect profile is as yet unknown, the tissue-specificity and the lower doses administered using our topical formulation are desirable in experimental studies and for future translational applications. Furthermore, while our genetic algorithm predicts the global optimal topical formulation for HA decrease based on the extrapolation of the results of the design of experiments, it is still somewhat based upon the concentration boundary parameters we predetermined and does not suggest a formulation that lies way outside of our predetermined range of concentrations. Thus, we conclude that a topical 4-MU formulation has experimental value, with an increased effect in females as shown by our innovative drug design schema, with a further need for optimization in a sex and possibly species-dependent manner.

Presently, defining a translational role for our topical formulation in human patients is beyond the scope of this study’s findings, but is certainly a future goal. Further experimental refinements are required in pursuit of that goal: at this time, the study’s limitations include the unpredictability of animal behavior and reaction to testing, and thus how it affected topical application of our experimental drug. All of the animals underwent their intervention at the same time, some even from the same litter, such that the effects of hair cycling and age differences were largely controlled. However, the issue of differing results with small shifts in the timing of these experiments (6 vs. 8 weeks), as described above, could also be more effectively controlled. In addition, we did not isolate the layers of the skin in some of our experiments, and thus the changes in HA content and HAS expression in response to topical 4-MU cannot be directly attributed to either the dermis or the epidermis. Further optimization of the topical 4-MU formulation would include differential, layer-specific targeting. A critical look at the components of the formulation may also be warranted, as it is known that EtOH can have a dose-dependent desiccant effect, possibly disruptive to the protective lipid matrix layer of the skin (Kownatzki 2003; Kwak et al. 2012). As we observed a notable sex-based difference in the in HA and HAS expression, both with and without 4-MU treatment, it would be critical to isolate the effects of sex hormones on HA and 4-MU in future experiments, which would be powered adequately to study sex as a biological variable. Moreover, upcoming experiments should involve determining molecular weight of HA in these skin samples, as the molecular weight of HA is currently considered a critical factor in its downstream effects (Garantziotis and Savani 2019).

In conclusion, this study details the process of designing an optimized topical 4-MU formulation that efficiently and effectively results in hyaluronan synthesis inhibition in normal mice skin. These findings provide a foundation for future study into, and treatment of, pathologic HA overexpression in common dermatologic disorders and malignancy.
